# Exertional Hemodynamics in Critical Care Cardiology: A Feasibility Study

**DOI:** 10.1016/j.chest.2025.08.025

**Published:** 2025-10-09

**Authors:** Aniket S. Rali, Hannah Granger, Christine Armstrong, Andrew DeFilippis, Marshall D. Brinkley, Kelly Schlendorf, Sandip K. Zalawadiya, JoAnn Lindenfeld

**Affiliations:** a Division of Cardiovascular Diseases, Vanderbilt University Medical Center, Nashville, TN; b Department of Anesthesiology, Division of Critical Care, Vanderbilt University Medical Center, Nashville, TN; c Department of Internal Medicine, Vanderbilt University Medical Center, Nashville, TN; d Department of Cardiac Rehab, Vanderbilt University Medical Center, Nashville, TN

To the Editor:

Hemodynamic assessment remains the most valuable adjunct to physical examination and laboratory evaluation in the diagnosis and management of cardiogenic shock. A myriad of hemodynamic monitoring devices, with varying degree of invasiveness, exist that rely on different physiological principles.^1^ Pulmonary artery catheter (PAC) has long been considered the gold standard of hemodynamic assessment in patients with cardiogenic shock and in recent years has been shown to improve clinical outcomes.^2^ While PAC provides comprehensive hemodynamic assessment at rest, it is inherently limited in assessing myocardial reserve with exertion in critically ill patients. Exertional hemodynamics are very valuable in confirming myocardial recovery and readiness to be weaned from inotropic as well as circulatory support in the critically ill.

VitalStream is a non-invasive, low-pressure finger sensor that measures BP, graphs pulse wave contour and subsequently uses pulse decompensation analysis (PDA) to estimate cardiac output (CO), cardiac power output (CPO) and systemic vascular resistance (SVR). Previous studies have validated VitalStream against invasive arterial lines in the cardiac catheterization lab and continuous thermodilution with a pulmonary artery catheter in patients undergoing cardiac surgery.^3^, ^4^, ^5^ We report a pilot study demonstrating the ease and feasibility of utilizing this device to assess advanced hemodynamics during ambulation in critically ill patients.

## Methods

Adult (≥ 18 years) patients admitted to the cardiac ICU (CICU) for treatment of cardiogenic shock and able to ambulate with physical therapy as part of their daily rehab were enrolled in this study after informed consent. Cardiogenic shock was diagnosed using hemodynamic assessment obtained during PAC and defined as central venous pressure ≥ 10 mm Hg, or pulmonary capillary wedge pressure ≥ 15 mm Hg and cardiac index < 2.2 L/min/m^2^ with hypotension (systolic BP < 90 mm Hg) and clinical signs of hypoperfusion. The study was reviewed and approved by Vanderbilt University Medical Center’s Institutional Review Board. Each patient had non-invasive BP, CO, CPO, stroke volume, and SVR measured at rest and throughout ambulation using VitalStream. All data obtained using VitalStream was for study purposes only and not utilized to make clinical decisions.

## Results

Twelve patients were approached and ultimately 10 were included in the analysis. All 10 patients were able to wear this device while ambulating without any discomfort or adverse impact. One patient experienced unstable ventricular tachycardia with ambulation, unrelated to the non-invasive device.

Continuous pulse contour, heart rate, and oximetry were displayed on a Bluetooth paired tablet which allowed the rehab team to monitor these variables in real time. Several patients required a walker to ambulate, and it did not significantly affect contact with the finger sensor or data collection. Furthermore, the device’s proprietary noise detection and cancelling algorithms were used to minimize unreliable data signal from being displayed on the monitor.

**Figure 1:**
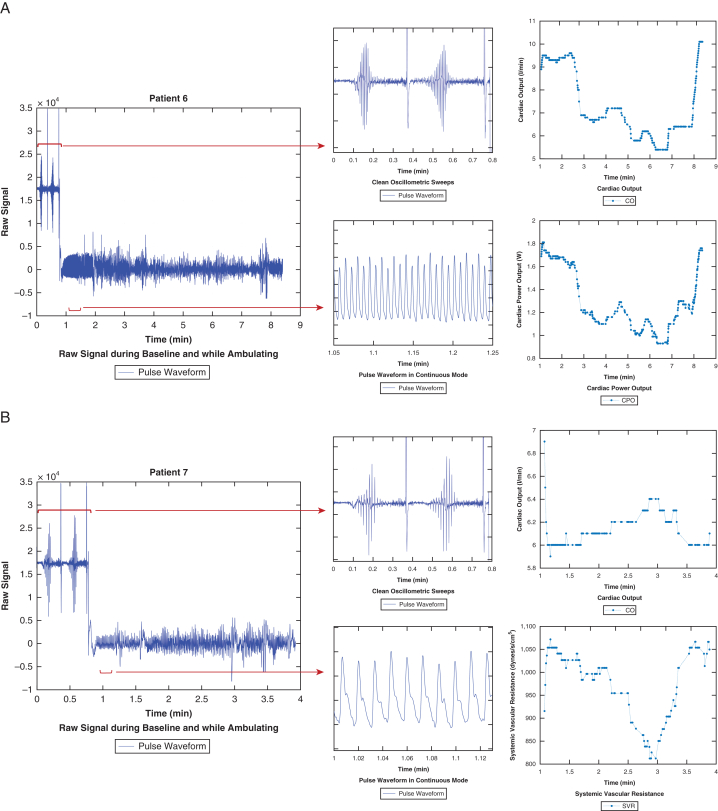

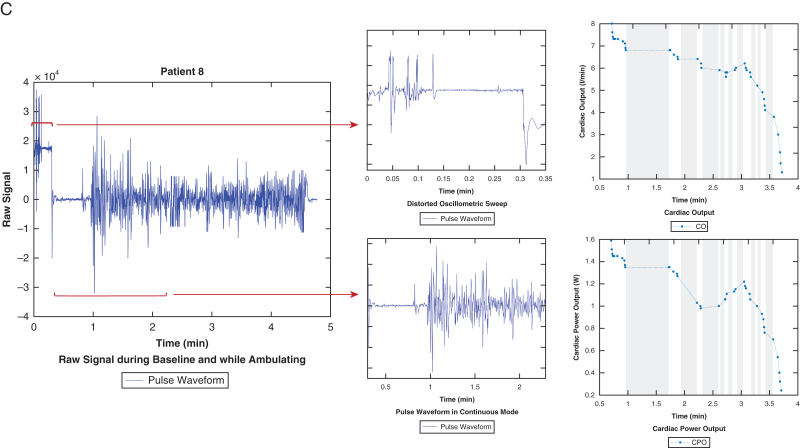
A, VitalStream data that were obtained from patient number 6 include the raw signal that highlights clean oscillometric sweeps and pulse waveforms in continuous mode. These waveforms were then analyzed by the device to report cardiac output and cardiac power output. B, VitalStream data that were obtained from patient number 7 include the raw signal that highlights clean oscillometric sweeps and pulse waveforms in continuous mode. These waveforms were then analyzed by the device to report cardiac output and systemic vascular resistance. Of note, cardiac power output was also reported but is not shown in this Figure. C, VitalStream data that were obtained from patient number 8 include the raw signal that highlights clean oscillometric sweeps and pulse waveforms in continuous mode. These waveforms were then analyzed by the device to report cardiac output and cardiac power output. Furthermore, times with low quality or inadequate signals are greyed out.

The majority of patients had strong pulse waveform signal obtained during baseline autocalibration as well as ambulation using pulse definition during oscillometric sweeps. In 2 patients, cardiac power output dropped with ambulation consistent with lack of myocardial reserve (example patient 6; Figure 1 A). In contrast, 4 patients demonstrated improvement in their cardiac output and cardiac power output during ambulation (example patient 7; Figure 1 B). Patient 9 initially demonstrated a rise in their cardiac output over the first 6 minutes of exertion but subsequently it dropped below resting baseline. Three patients had instances of poor pulse pressure signal despite repeated attempts at recalibration and hence had significant number of missing measurements (example patient 8; Figure 1 C).

## Discussion

The key findings of our feasibility study using the VitalStream device are as follows: 1) In patients with cardiogenic shock, VitalStream can be used with relative ease to obtain hemodynamics including CO and CPO. 2) The data obtained from this device may help evaluate myocardial reserve with exertion.

This study demonstrated very different hemodynamic profiles of patients who were able to augment their CO during ambulation compared to those who were not. Such an assessment of myocardial reserve in critically ill patients has historically been limited due to the invasive nature of continuous hemodynamic monitoring devices. Future studies will be required to confirm the validity of non-invasive measurements in patients with cardiogenic shock and assessing the impact of exertional hemodynamic assessments on patient outcomes including successful weaning of circulatory support or inotropes, ICU length of stay, and ICU readmissions.

Additionally, given the non-invasive nature of VitalStream device, it may allow for sequential assessment of advanced hemodynamics after transfer out of the ICU. This would be especially important to monitor for continued improvement as well as early recognition of recurrent decompensation. A major advantage of the VitalStream device is that the hemodynamic assessment, in various clinical settings, could be performed using the same device, eliminating inter-device variability. While VitalStream has previously been validated against continuous thermodilution PAC in patients undergoing cardiac surgery,^3^ it largely remains unknown how accurate the device is in patients with very low CO. Additionally, it is unclear if the accuracy of this device can be further improved by machine learning algorithms in patients with simultaneous PAC and VitalStream measurements.

In conclusion, our results suggest that it is feasible to use the VitalStream device in ambulatory critically ill patients to obtain advanced hemodynamics

## Financial/Nonfinancial Disclosures

The authors have reported to *CHEST* the following: A. S. R. has received consulting honorarium from CareTaker Medical, Abiomed, Analog Devices and Zoll. J. L. has received consulting honoraria from Abbott, Abiomed, Adona, Alleviant, AskBio, AstraZeneca, Axon, Boston Scientific, Cordio, CVRX, Edwards Lifesciences, Fire 1, Intershunt, Johnson and Johnson, Medtronic, Merck, OrchestraBiomed, Pulnovo, VWave, Vascular Dynamics, Whiteswell, Windmill Cardiovascular Systems, and Volumetrix. A. D. has received consulting honorarium from NovoNordisk and Velakor Inc, and research grants from NIH and AHA. The remaining authors have nothing to disclose. None declared (M. D. B., K. S., S. K. Z., H. G., C. A.).
